# Asymmetric Stochastic Switching Driven by Intrinsic Molecular Noise

**DOI:** 10.1371/journal.pone.0031407

**Published:** 2012-02-21

**Authors:** David Frigola, Laura Casanellas, José M. Sancho, Marta Ibañes

**Affiliations:** Department of Estructura i Constituents de la Matèria, Facultat de Fsica, Universitat de Barcelona, Barcelona, Spain; University of Maribor, Slovenia

## Abstract

Low-copy-number molecules are involved in many functions in cells. The intrinsic fluctuations of these numbers can enable stochastic switching between multiple steady states, inducing phenotypic variability. Herein we present a theoretical and computational study based on Master Equations and Fokker-Planck and Langevin descriptions of stochastic switching for a genetic circuit of autoactivation. We show that in this circuit the intrinsic fluctuations arising from low-copy numbers, which are inherently state-dependent, drive asymmetric switching. These theoretical results are consistent with experimental data that have been reported for the bistable system of the gallactose signaling network in yeast. Our study unravels that intrinsic fluctuations, while not required to describe bistability, are fundamental to understand stochastic switching and the dynamical relative stability of multiple states.

## Introduction

Stochastic fluctuations are ubiquitous in any real dynamical system: physical, chemical, biological, etc. In particular, living organisms are subject to fluctuations (or noise) of distinct origins. At the cellular level, it is a well known fact that biochemical reactions inside a cell are discrete and stochastic events and present inherent randomness. This randomness is more evident when the molecules involved in the dynamical process are present in small numbers. These fluctuations can have disturbing or ordering roles.

Recently, it has been shown that cells may exploit noise in different beneficial ways. For instance, noise may act as a trigger for phenotypic variability since fluctuations enable the exploration of the phase space through different types of dynamics ([1]–[5], for reviews). This has been observed in several natural systems, like in the gallactose utilization network in the budding yeast [6], the process of DNA uptake from the environment in *B. subtilis* [7], [8], photoreceptor differentiation in the fruit fly retina [9] and in stem cell differentiation [10]–[12]. Since the roles and benefits of stochastic phenomena in natural systems are starting to be elucidated, it becomes relevant to characterize thoroughly the features of such stochastic phenomena in terms of the driving fluctuations.

In bistable or multistable systems, variability or phase space exploration can occur through stochastic switching, *i.e.* the random transition from one state to another one, and it has been shown to be beneficial for isogenic populations in changing environments [13], [14]. Well known examples of bistable systems are biochemical switches which have two stable solutions corresponding to high and low (ON/OFF) concentration states [15]. Genetic switches have been reported abundantly in natural systems (see [16]–[20] for some examples) and have been constructed synthetically as well [21]–[24]. Commonly, these switches arise from nonlinear dynamics involving a positive feedback loop in which a molecular species upregulates, directly or indirectly, its own production.

In biochemical bistable systems stochastic switching becomes more probable when the bistable states have little enough differences in copy numbers [25]–[28]. This switching enables phenotypic variability but prevents stable memory of past history [6]. Experimentally, both bistability and hysteresis have been reported for several stable switches [6], [29], [30]. The dependence of hysteresis (or memory of past history) and stochastic switching on circuit architectures such as positive and negative feedbacks has been evaluated both experimentally and theoretically [6], [31]. Importantly, the natural system of the gallactose signaling network in yeast has been driven to a regime showing frequent enough stochastic switching and its rates have been measured [6].

Herein we address the issue of how intrinsic noise modulates stochastic switching rates. To this end, we use one of the simplest descriptions of a biochemical bistable switch which corresponds to autoactivation. In this case, a single molecular species describes the switch and its nonlinear dissipative dynamics can be related to overdamped dynamics on an energy potential [22], [32]. In order to characterize stochastic switching dynamics in this circuit, the most appropriate theoretical scenario to be used is the Master Equation since it incorporates in a natural way the presence of intrinsic fluctuations. We use as well the corresponding Fokker-Planck equation since it enables the theoretical evaluation of the switching rates. In order to pinpoint the dynamical features introduced just by intrinsic noise, we make a comparison with a second model using the Langevin dynamics formalism. In this latter model, fluctuations arise instead from a thermal bath, *i.e.* from non-intrinsic, uniform noise. Altogether, our study characterizes the dependence of steady and dynamical properties of autoactivation on intrinsic noise.

## Methods

### 1 Deterministic description

We have used a simple chemical kinetic model for autoactivation commonly used in the literature (see [15], [22], [31] for instance). In this autoactivation circuit, a protein promotes its own production according to a Hill function with cooperativity 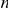. Since usually mRNA degrades more rapidly than protein, we consider mRNA dynamics to be much faster than protein dynamics (quasi-steady state approximation) and use a single equation, which describes the protein dynamics. The deterministic dynamic equation for such a system is
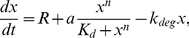
(1)where 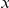 denotes the concentration of protein, 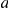 is the maximum production rate, 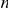 represents cooperativity, 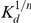 sets the value at which the production rate is half its maximum value, 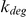 is the degradation rate and 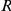 is the basal production rate. We can rewrite this equation with dimensionless variables in such a way that the least possible parameters are left:
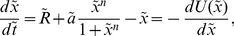
(2)where
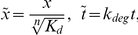


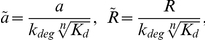
and 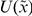 is the energy potential, which for 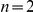 reads:
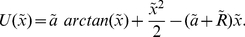
(3)For this dimensionless dynamics, 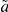 has been used as control parameter. This deterministic description as described above is independent of the cell volume 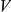. However, when this framework is related to stochastic kinetic reactions, the dependence on the cell volume becomes evident. Accordingly, and for the sake of compactness, herein we introduce the parameter values from [31]: 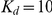 nM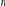, 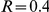 nM min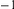, 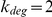 min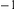, and 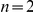. In order to satisfy 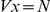, where 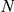 is the number of molecules, then the dimensionless cell volume shall be 
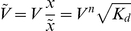
, which, using the value 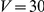 nM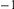, is 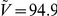. All the study has been performed using the dimensionless variable and parameters. For the sake of simplicity, hereafter these dimensionless variable and parameters are not indicated with tilde or superindex.

### 2 Stochastic description I: Multiplicative noise model

When the molecular species are present in small numbers, the stochasticity of chemical reactions becomes more evident and the deterministic description no longer describes accurately the real dynamics. A stochastic description is then required. Biochemical reactions can be described by birth-death processes governed by chemical master equations [28]. To model the autoactivation circuit dynamics we have considered two transition processes 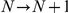 and 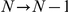 with rates, following [31], given by, respectively,
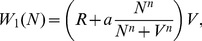
(4)

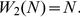
(5)



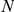
 stands for the number of molecules and 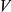 for the nondimensional cell volume properly adjusted for the dimension transformation described above. The corresponding Master equation [33] is
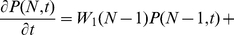
(6)

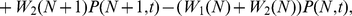
where 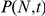 is the probability distribution at time 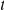.

We have simulated numerically this Master equation dynamics with the Gillespie algorithm [34] with custom-made software.

Rewriting the Master equation with concentration (continuous) variables, the corresponding Fokker-Planck equation [25], [35] for the system is obtained
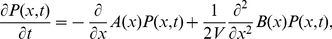
(7)

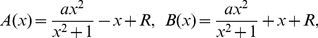
(8)where 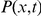 is the probability of having a concentration 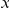 at time 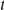. The Fokker-Planck equation is amenable to theoretical stochastic analysis. This equation can be readily solved in the stationary regime [35], obtaining the steady state probability
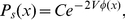
(9)where 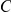 is a normalization constant and 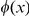 is the effective stochastic potential (as opposed to the deterministic potential in Eq (3))
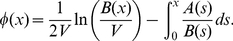
(10)


An equivalent description to the Fokker-Planck equation, which provides actual stochastic trajectories as opposed to probability distributions, is the Langevin equation. The Langevin equation corresponding to Eq (7) in the Itô interpretation [33] is
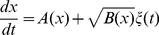
(11)where 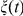 is a Gaussian white noise with
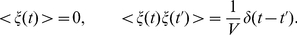
(12)This corresponds to the so-called chemical Langevin equation [36]. This description identifies 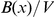 with the square power of the noise intensity. The noise becomes reduced as the cell volume 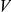 increases. For 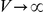, we recover the deterministic description of Eq (2).

Notice that the noise term appears in the Langevin equation with a state-dependent term, 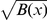, multiplying it. Therefore, the intrinsic noise coming from the biochemical reactions arises naturally in this equation as a multiplicative noise. Hereafter we call this dynamics (either in the Langevin, Fokker-Planck or Master equation description) as the multiplicative noise scenario.

Like the Master equation, the Langevin description enables the time-integration of the dynamics, obtaining simulated stochastic trajectories. In contrast with the Master equation description, the Langevin approach focuses on a continuous variable, the concentration of the molecular species. We have numerically integrated this Langevin equation with custom-made software using the algorithm in [37].

### 3 Stochastic description II: Additive noise model

For comparison, we have studied also the states and dynamics of a description that takes constant noise regardless of the protein concentration 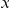. This corresponds to analyze the autoactivation circuit in a thermal bath. It does not correspond to a description based on the stochastic chemical equations and the noise term does not account for intrinsic fluctuations. Instead, this is a description which has been commonly used in the study of genetic circuits to introduce fluctuations as a mere jiggling of the steady states, without taking into account the origin of this randomness.

We have constructed this dynamics from the Langevin equation by setting the deterministic dynamics plus a noise term which is state-independent:
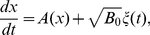
(13)with 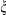 given by Eq (12). Notice that the difference with the multiplicative noise scenario relies on the use of 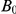, a constant, instead of the function 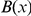. Hereafter we call this approach the additive noise case, since the noise enters in an additive way. The stationary solutions and the bifurcation diagram of this model are the same as for the deterministic model.

The Fokker-Planck equation corresponding to the above Langevin equation (13) reads:
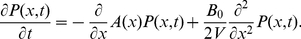
(14)From this Fokker-Planck equation we can obtain the stochastic potential for the additive case, 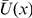, which is proportional to the deterministic energy potential 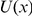 up to shift and scale factors:
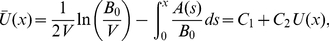
(15)where 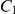 and 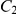 are constants given by 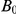 and 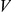. Accordingly, the relative stability of the states provided by this function is the same as the one derived from the energy potential 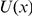.

For a good comparison between the additive and multiplicative noise cases, we have chosen a value of 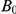 such that the stochastic potential 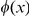 and the potential 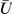 coincide at the OFF state value of the multiplicative noise dynamics. For each 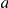, a 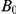 value can be evaluated. However, we have observed no significant differences if a common value of 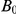 is used for any 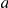. Thus in all figures, unless indicated otherwise, we have used 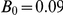 which corresponds to 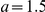.

We have simulated stochastic trajectories of the concentration 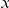 from the Langevin equation using the Heun algorithm [38] and, to avoid unrealistic negative values of 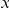, a reflecting boundary at 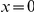 has been introduced.

### 4 Mean First Passage Time (MFPT)

The MFPT gives the average time to switch from one state to another one. The MFPT, 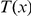, satisfies the following differential equation [33]

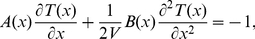
(16)which can be solved with the proper boundary conditions: an absorbing boundary at the maximum and a reflecting boundary either at 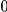 or 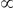 ,
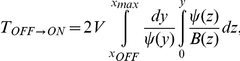


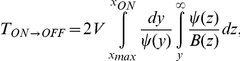
where
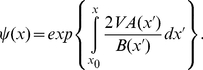
(17)with 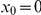 for the 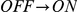 transition, and 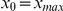 for the 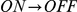 transition.

The theoretical results concerning the MFPT have been obtained by calculating numerically (using a Romberg algorithm [39]) these expressions for both the multiplicative and the additive noise cases (in the additive noise case, 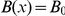 has been used).

We have also obtained the MFPT from simulations of the stochastic trajectories of the number of molecules, obtained from the Master equation, and of the concentration, obtained from the Langevin equations by simulating trajectories around each stable state, measuring how long do they take to cross the maximum of the potential for the first time and averaging this value for 100 to 500 repetitions of the same process (which just differ in the stochastic numbers).

## Results

### 1 Bistability

It is well known that positive feedback loops formed by autoactivation exhibit bistability. Specifically, both the deterministic and stochastic models presented in sections 1 and 2 respectively in Methods have been shown to have a bistable regime [22], [31]. However, no detailed comparison of both descriptions has been performed yet, as far as we know. In this section we are interested in evaluating the effect of intrinsic fluctuations in the steady states. Accordingly, we compare the bifurcation diagrams for the stochastic multiplicative noise and for the deterministic models. From a biophysical point of view, by doing so we are comparing the features of the same autoactivation circuit in two cells with very different volumes. The autoactivation circuit in the cell with a small volume would be described by the stochastic multiplicative noise model, whereas it would be well approximated by the deterministic description in the cell with a very (extremely) large volume.

**Figure 1:**
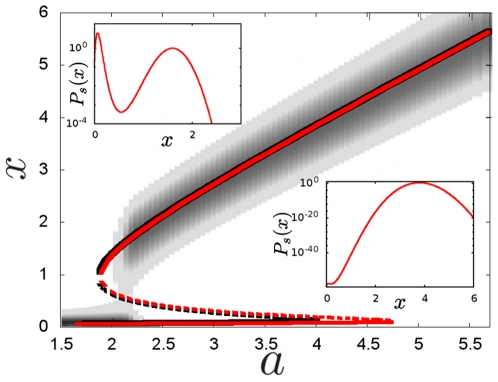
Steady state values do not change significantly when intrinsic noise is included. Bifurcation diagram for the deterministic model (black) and the multiplicative noise model (red). Stable steady states (continuous lines) and unstable steady states (dashed lines) are minima and maxima, respectively, of the potentials. The bifurcation diagram of a stochastic description with a thermal bath (additive noise) is necessarily the same as the one of the deterministic model. The stationary probability distribution for the multiplicative noise model, Eq (9), for different 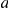 values is shown in grey scale. Insets: Stationary probability distributions for the multiplicative noise model for 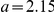 (top) and 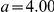 (bottom).

The bifurcation diagram for the control parameter 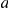, related to the maximal molecular production rate, is shown in Fig. 1. The steady state solutions of the bifurcation diagram have been obtained by computing numerically (Mathematica Software [41]) the minima and maxima of the potentials, Eqs (3) and (10), for the deterministic and stochastic models. As it is shown, both descriptions show a very similar bifurcation diagram with a bistable regime for intermediate values of 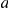 in which two stable states, a low-concentration state (OFF) and a high-concentration (ON) state, can coexist. The steady state concentrations are very similar among the two descriptions. A difference among the bifurcation diagrams is an enlargement of the bistability region for the stochastic multiplicative noise model. However, when stochastic switching between the states is taken into account, this enlargement becomes not relevant. Indeed, for this region, it is extremely easy to escape from the OFF state and to switch (irreversibly, for very long time scales) to the ON state [31]. Hence, bistability is not expected to be observed in this region precluding the observation of differences between the deterministic and the stochastic descriptions (compare insets in Fig. 1). In fact, bistability is especially obvious in a narrow region (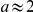) which is common to both descriptions (see grey areas in the figure).

Our results show that stable steady state concentrations do not depend strongly on intrinsic fluctuations. These results indicate that bifurcation diagrams of autoactivation circuits obtained experimentally can be fitted appropriately just by the deterministic description of the dynamics.

### 2 Fluctuations

**Figure 2:**
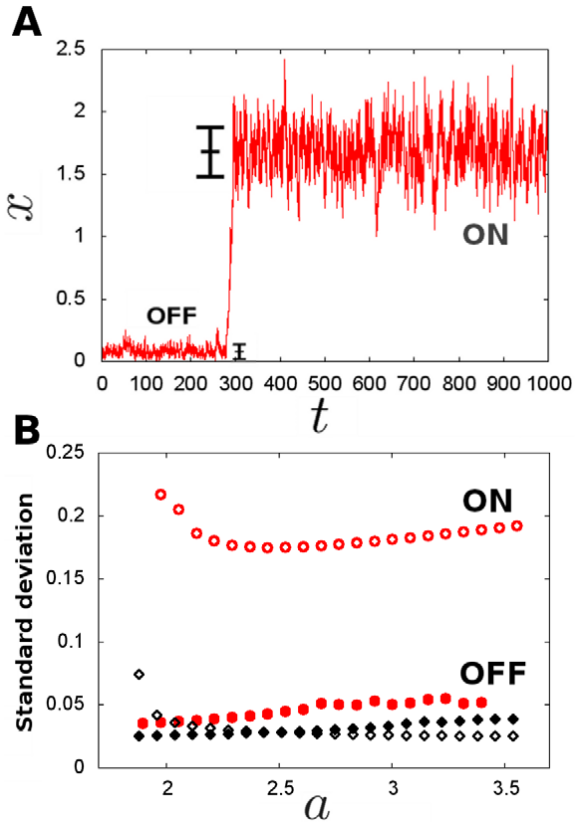
Intrinsic multiplicative noise drives larger absolute fluctuations in the ON state. **A** Time evolution of the concentration 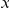 for 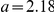. Error bars denote the fluctuations size in each state, which is much higher in the ON state. **B** Fluctuations in the OFF steady state (filled symbols) and in the ON steady state (empty symbols) for the multiplicative noise model (red) and for the additive noise model (black). Fluctuations are measured as the standard deviation from the steady state. For the multiplicative noise model, fluctuations in the ON state are larger than in the OFF state. For the additive noise model, fluctuations in the OFF and ON states are similar. Standard deviations have been computed over samples of sizes ranging from 100 to 1000 repetitions of the corresponding Langevin dynamics at time t = 100. For the additive noise model we have used a different 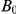 value for each 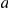, as explained in section 3 in Methods.

Intrinsic stochasticity of the biochemical reactions of the autoactivation circuit result in state-dependent multiplicative noise (see section 2 in Methods). Fluctuations are expected to be larger in the ON state than in the OFF state (Fig. 2A) because the noise intensity increases with the concentration according to the function 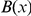. Since dynamics such as MFPTs depend on absolute fluctuations we have computed the standard deviation of concentrations in each stable steady state. Numerical simulations of the stochastic multiplicative noise dynamics show that for all the bistable region, absolute fluctuations are larger in the ON state than in the OFF state (Fig. 2B).

The coefficient of variation (*i.e.* relative fluctuations, defined as the standard deviation over the mean) is larger in the OFF state and decreases for larger volumes, as expected (see Table 1) [40]. However, it is important to notice that the coefficient of variation is not the relevant magnitude in our analysis as we will show below.

**Table 1:** The ratio between fluctuactions in the OFF and ON states is mantained at larger volumes.

	OFF	ON	OFF/ON
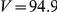	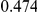	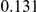	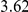
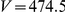	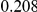	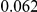	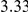

Relative fluctuations in each steady state for two different nondimensional cell volumes 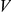 for 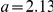 for the multiplicative noise model. Relative fluctuations have been computed as the ratio between the standard deviation over the mean steady state. Standard deviations and mean values have been extracted from Langevin dynamics as in Fig. 2. As shown, relative fluctuations decrease with cell volume, but are always larger in the OFF state. The ratio between the relative fluctuations in the two states is indicated in the last column. This ratio is little sensitive to the cell volume.

Fluctuations in an energy potential well depend on the shape of the potential. Since the energy potential corresponding to autoactivation dynamics is asymmetric we can expect the ON and OFF states to exhibit different standard deviations even if the noise intensity is the same in both cases. To ensure that the differences in standard deviation observed in Fig. 2B are driven by intrinsic noise and are not just the result of an asymmetric energy potential, we computed the standard deviation for each steady state for an additive noise model (see section 3 in Methods). In this additive noise model, the noise intensity is the same for all states and the dynamics are subjected to the energy potential of autoactivation. Note that noise in this additive noise model stands for a thermal bath and not for intrinsic fluctuations. As shown in Fig. 2B, fluctuations in the additive noise model are very similar in the OFF and ON states. This result indicates that the asymmetry of the energy potential does not drive a significant difference in the fluctuations around each steady state, and thus is not responsible for the large differences observed in the multiplicative noise model with intrinsic noise.

Altogether we have shown that intrinsic noise in the positive feedback loop of autoactivation creates larger absolute fluctuations in the ON state than in the OFF state.

### 3 Stochastic switching

**Figure 3:**
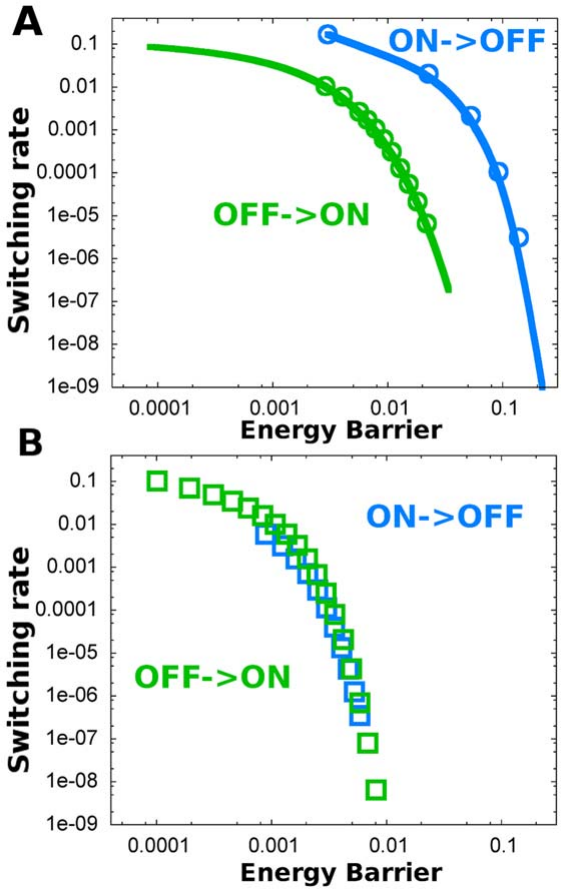
Intrinsic multiplicative noise generates an asymmetry in switching rates. **A** Switching rate versus energy barrier for the stochastic system with intrinsic multiplicative noise. The lines represent the values obtained through theoretical MFPT calculations, Eq (16), and the circles represent the values obtained through simulation (Gillespie and Langevin are identical). In both panels, the energy barriers were calculated from Eq (3). Blue colour corresponds to switching from ON to OFF and green color corresponds to OFF to ON switching. **B** Switching rate versus energy barrier for the additive noise case. Notice how the rates for both states keep the same relation with the energy barriers. Colour code is as in previous panel. Symbols correspond to theoretical MFPT calculations. Simulations are in perfect agreement, but are not represented for clarity. In both panels, the nondimensional cell volume is 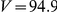.

Stochastic switching dynamics depend on the energy potential and on fluctuations. Since intrinsic noise drives different fluctuations in the ON and OFF states we may expect different switching dynamics from each state. To evaluate the role of intrinsic noise on the switching dynamics, we measured the escape or switching rates as the inverse of the MFPT (see section 4 in Methods) for the multiplicative noise dynamics. When plotting these rates as a function of the energy barrier (Fig. 3A), we see that the switching becomes asymmetric: for the same energy barrier height, it is more probable to switch from the ON state than from the OFF state.

To corroborate whether this asymmetry is driven by intrinsic noise, we measured the escape rates for the additive noise model. For this model, the asymmetric effect is absent (Fig. 3B). Together, our results show that state-dependent intrinsic noise in autoactivation dynamics drive an asymmetric switching.

**Figure 4:**
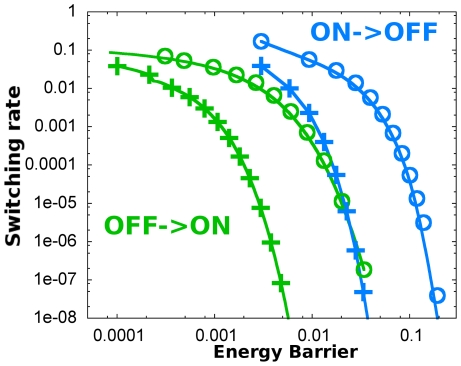
The asymmetry of switching rates does not disappear at larger volumes. Switching rates for different cell volumes as a function of the energy barrier. Switching rates (computed from Eq (16)) of the stochastic system with intrinsic multiplicative noise for an adimensonal cell volume 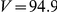 (circles) and for a larger volume 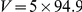 (crosses). The energy barrier heights were calculated from Eq (3). Blue color corresponds to ON to OFF switching and green corresponds to OFF to ON switching. The asymmetry of the switching rates is observed for both volumes.

Importantly, the differences in fluctuations among the ON/OFF states arising from intrinsic noise are preserved for different cell volumes and are little sensitive to changes in the cell volume (Table 1). Hence, we can expect that the phenomenology of asymmetric switching rates holds for a wide range of cell volumes. Fig. 4 shows this is indeed the case. For larger cell volumes the switching rates decrease overall (since the switch becomes more stable [25]), but they still show a similar relative asymmetry. It is still more probable to switch from the ON state than from the OFF state for equal energy barrier height values. This result stresses the importance of intrinsic fluctuations at a fundamental level.

**Figure 5:**
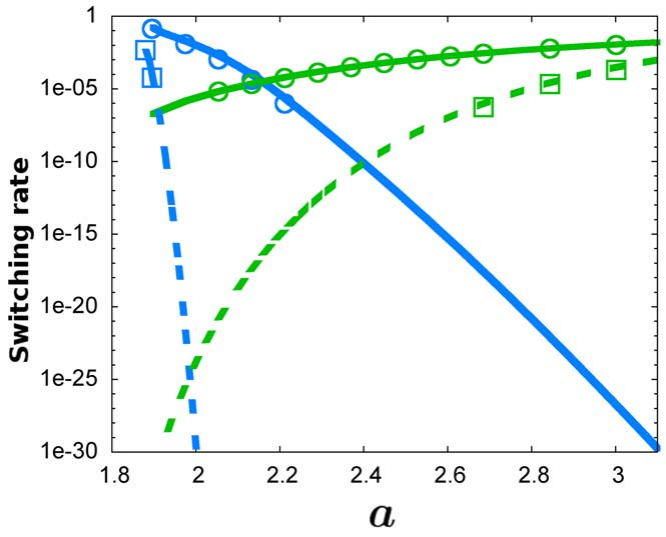
Intrinsic multiplicative noise increases the domain where the OFF state predominates. Switching rates for stochastic transitions from ON to OFF (blue) and viceversa (green) for the additive (dashed lines) and for the multiplicative (continuous lines) systems, computed from Eq (16). The critical value 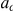 at which the switching rates for the two transitions are equal is shifted from 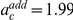 in the additive case to 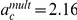 for the multiplicative noise scenario. Results for Langevin simulations of the additive noise model (squares) and for Gillespie simulations of the multiplicative noise model (circles) are depicted.

The asymmetry can be also observed in the value of 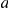 at which the switching rates from the OFF states and from the ON states are the same [31]. This value is larger when intrinsic noise is taken into account (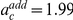, 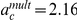, see Fig. 5). This shift indicates that intrinsic fluctuations enlarge the control parameter 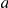 region for which it is less frequent to switch from the OFF state than from the ON state.

Our results show that intrinsic fluctuations in autoactivation dynamics introduce a state-dependent noise which consistently drives larger absolute fluctuations in the ON state and elicit a faster switching rate from this state than from the OFF state for the same energy barrier height. Remarkably, this type of asymmetry is in agreement with the one reported experimentally for the gallactose signaling switch in yeast cells [6]: it is more probable to switch from the ON to the OFF state than viceversa.

### 4 Relative stability of steady states

It is worth here to comment the previous results on asymmetric switching and the role of intrinsic noise on the relative stability between states. To this end, we compared the stochastic potential of the multiplicative noise model Eq (10) with the energy potential Eq (15). Note we used Eq (15) which is, up to scale and shift factors, the deterministic energy potential Eq (3). We set 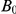 to match the potentials at the OFF state (see section 3 in Methods), facilitating their comparison.

**Figure 6:**
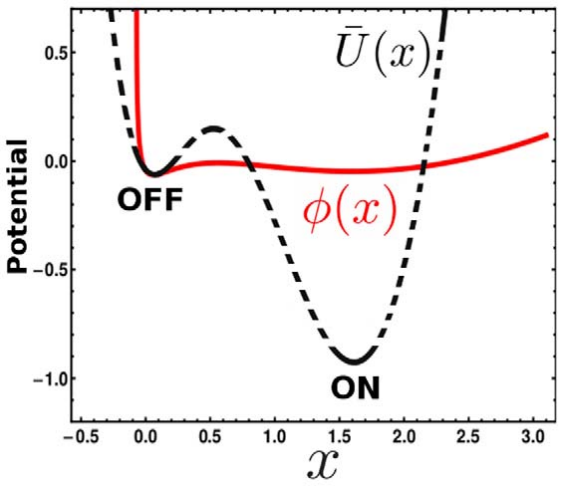
Intrinsic multiplicative noise changes the relative stability of the ON state. Stochastic potential of the additive noise model (black dashed line, Eq (15)) and of the multiplicative noise scenario (red continuous line, Eq (10)) for 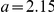. As shown, the ON state is clearly destabilized by multiplicative noise. This is also observed for different values of 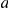.

The two potentials are shown in Fig. 6. The stochastic potential for the multiplicative noise scenario has been previously reported in [31]. As shown in Fig. 6, the multiplicative noise affects drastically the ON state, reducing the barrier height and decreasing the curvature of the potential at the ON state. Moreover, the fact that the well potential in the ON state becomes flattened due to the intrinsic multiplicative noise implies larger fluctuations in the copy number which, in turn, will induce faster transitions. These two changes favor the transition rate from the ON state to the OFF one, thus reducing the stability of the ON state.

These results show that inherent and intrinsic fluctuations in a positive feedback loop based on autoactivation drive large changes which could reduce the differences in the stability of the steady states. Hence, the relative stability of the bistable states is a dynamical phenomenon which is very sensitive to the noise characteristics.

## Discussion

We have presented a theoretical and numerical analysis of the role of intrinsic noise in a bistable switch with autoactivation dynamics. Our theoretical approach is consistent and independent of a particular scenario either using Master or Langevin equations, and is complemented with numerical integrations and stochastic simulations. Our results exemplify that intrinsic noise in autoactivation dynamics, which result in multiplicative noise (state-dependent fluctuations), are a relevant ingredient for the dynamics but not for the characterization of the steady states. Specifically, while the bifurcation diagram is mostly unchanged when intrinsic noise is taken into account, the switching dynamics and the relative stability of the states are very sensitive to state-dependent fluctuations.

Recently it has been shown that noise can be different in the ON and OFF states of feedforward loop genetic circuits [42]. For a genetic circuit involving positive and negative feedbacks it has been also shown that intrinsic noise can stabilize a deterministically unstable state [43]. Herein, we show that intrinsic noise in autoactivation dynamics drives the ON state less stable. Specifically, intrinsic noise drives larger absolute fluctuations in the ON state which elicit a faster switching rate from this state than from the OFF state for the same energy barrier height. Remarkably, this phenomenology holds for different cell volumes, and accordingly for different noise intensities. We have termed this phenomenon asymmetric stochastic switching.

Asymmetric stochastic switching has been observed in the gallactose signalling network in yeast [6]. In this network, a positive feedback loop involving the cytoplasmic molecule Gal3p drives bistability of low (OFF) and high (ON) pathway activity states in which GAL3 expression is low and high respectively [6]. For a specific parameter regime, yeast cells can switch spontaneously and stochastically between these states during the time period being analyzed. When comparing the switching rates from each (OFF/ON) state for the same value of the energy barrier height, Acar et al. obtained that it is more probable to switch from the ON to the OFF state than viceversa [6]. Moreover, they measured the fluctuations of GAL3 expression in each state and concluded that fluctuations are larger in the ON state than in the OFF state [6]. These two features, larger probability of switching from the ON state and larger fluctuations in the ON state, are analogous to the ones we obtain by theoretical and numerical means for the stochastic autoactivation switch with intrinsic noise.

Together, our study explains that although the bistability phenomenon is rather independent of the noise characteristics, the relative stability of each state and stochastic switching dynamics are dynamical features very sensitive to the kind of noise: additive or multiplicative. A simplistic approach with an additive noise can not address all the possible phenomenologies and one has to resort to carefully considering noise as an intrinsic part of the system, which is relevant at a fundamental level and not as a correction.
